# Effect of Pluronic-F68 fog solution on performance and morbidity of newly received heifer calves

**DOI:** 10.1093/tas/txaa124

**Published:** 2020-12-22

**Authors:** Consuelo A Sowers, Vinicius N Gouvêa, Michael L Barnes, Glenn C Duff

**Affiliations:** Clayton Livestock Research Center, New Mexico State University, Clayton, NM

**Keywords:** health, heifers, performance, Pluronic-F68

## Abstract

Morbidity and mortality from bovine respiratory disease (BRD) of newly received feedlot cattle continue to be problems for the feedlot industry. The objective of this study was to evaluate the effects of utilizing a novel breathing treatment containing a nonionic surfactant (Pluronic-F68) on performance and morbidity of high-risk calves during a 45-d receiving period. Angus/Angus-cross heifer calves (*n* = 240) were acquired in Delhi, LA, and transported (14 h) to the research facility. Heifers were allowed 21-h rest with access to water and RAMP prior to processing. Heifers were sorted into 16 pens by processing order and randomized by pen into one of two treatments: novel breathing treatment containing 6.25% Pluronic-F68 solution, 28.13% glycerin, and 65.62% water (FOG; *n* = 8 pens per treat and 15 heifers per pen) and control (CON; *n* = 8 pens per treat and 15 heifers per pen). Control heifers were held in an enclosed stock trailer for 10 min and followed by FOG heifers, during which time treatment was administered. The person responsible for identifying signs of morbidity was blinded to treatment assignments. Data were analyzed as a completely randomized design using MIXED (continuous) or GLIMIX (binomial) models of SAS 9.4. Average daily gain was similar between treatments (*P* = 0.91). No differences were found in dry matter intake (*P* = 0.14) nor in gain efficiency (*P* = 0.58). There were no differences (*P* = 0.74) in final body weights. Morbidity was similar at first, second, and third antimicrobial administration regardless of treatment (*P* ≥ 0.34). The number of antimicrobial treatments required or the management of BRD was similar between treatments (*P* = 0.72). There was no difference (*P* = 0.44) in mortality between FOG and CON groups. The Pluronic-F68 solution did not improve performance or reduce morbidity of newly received heifer calves; however, further research with a different concentration and/or duration of fogging may be warranted.

